# Plasma Cortisol and Risk of Atrial Fibrillation: A Mendelian Randomization Study

**DOI:** 10.1210/clinem/dgab219

**Published:** 2021-04-05

**Authors:** Susanna C Larsson, Wei-Hsuan Lee, Stephen Burgess, Elias Allara

**Affiliations:** 1 Unit of Cardiovascular and Nutritional Epidemiology, Institute of Environmental Medicine, Karolinska Institutet, Stockholm, Sweden; 2 Unit of Medical Epidemiology, Department of Surgical Sciences, Uppsala University, Uppsala, Sweden; 3 BHF Cardiovascular Epidemiology Unit, Department of Public Health and Primary Care, University of Cambridge, Cambridge, UK; 4 MRC Biostatistics Unit, University of Cambridge, Cambridge, UK

**Keywords:** Atrial fibrillation, Cushing’s syndrome, cortisol, Mendelian randomization

## Abstract

**Context:**

Atrial fibrillation (AF), cardiac arrhythmias, and related risk factors are common in patients with Cushing’s syndrome, or clinical chronic hypercortisolism. While hypercortisolism may be associated with AF, this association has not yet been ascertained causally.

**Objective:**

To determine whether plasma cortisol is causally associated with AF using a 2-sample Mendelian randomization (MR) design.

**Methods:**

Three genetic variants in the *SERPINA1/SERPINA6* locus and functionally associated with plasma cortisol were identified in the CORtisol NETwork consortium (12 597 participants). Summary-level genome-wide association study (GWAS) data for the associations between the cortisol-associated variants and AF were obtained from a GWAS meta-analysis of 6 studies (60 620 AF cases and 970 216 noncases) and the FinnGen consortium (17 325 AF cases and 97 214 noncases). The fixed-effects inverse-variance weighted approach accounting for genetic correlations between variants was used for analysis. Multivariable MR analyses were conducted to assess potential mediating effects of systolic blood pressure (SBP) and waist circumference (WC). Summary-level GWAS data for SBP and WC were obtained respectively from the International Consortium of Blood Pressure (757 601 participants) and the Genetic Investigation of ANthropometric Traits consortium (232 101 participants).

**Results:**

One standard deviation increase in genetically predicted plasma cortisol was associated with greater risk of AF (odds ratio [OR] 1.20, 95% CI 1.06-1.35). The association attenuated when adjusting for genetically predicted SBP and WC (OR 0.99, 95% CI 0.72-1.38).

**Conclusion:**

Evidence derived from the MR study suggests a positive association between plasma cortisol and risk of AF, likely mediated through SBP and WC.

Cortisol is a steroid hormone that is vital in the response to stress and regulates a wide range of homeostatic functions in the human body. Unregulated cortisol levels can contribute to metabolic pathophysiology (1). Abnormally high levels of cortisol for a prolonged period of time can lead to Cushing’s syndrome (CS). Common clinical manifestations of CS include hypertension, obesity, and hypokalemia (2-4), all of which are metabolic risk factors for atrial fibrillation (AF), the most common sustained arrhythmia. Observational evidence also suggest that AF is likely in patients with CS (5).

Since patients with CS have cortisol levels at the extreme end of the population distribution, common features of CS, also risk factors for AF, can approximate potential outcomes of prolonged exposure to high levels of cortisol in otherwise healthy populations. While the connection between stress and cortisol secretion is well known (6) and the association between stress-induced hypercortisolemia and AF has been investigated in animal tissue studies (7), human observational studies have not yet investigated the association between cortisol and AF in the general population. However, large cohort studies have provided evidence supporting potential association between exposure to external stressors and AF (8, 9).

Importantly, limitations inherent to observational designs, such as confounding and reverse causality, preclude causal interpretation of the potential association between hypercortisolemia and AF both in patients with CS and in the general population. To clarify the potential causal association between physiologically high cortisol levels and risk of AF, we conducted a Mendelian randomization (MR) study using data from large-scale genetic consortia. We assessed potential mediating effects by systolic blood pressure and central obesity (measured as waist circumference). Understanding whether cortisol is causally associated with AF can provide insight into treatment and monitoring of cardiovascular-related CS symptoms and reduce CS morbidity. Additionally, this analysis may provide indirect evidence as to whether prolonged hypercortisolemia can impact AF incidence in the general population.

## Materials and Methods

### Data sources

Morning plasma cortisol was proxied by 3 single nucleotide polymorphisms (SNPs), rs2749527, rs12589136, and rs11621961, in *SERPINA6* (encoding corticosteroid binding globulin) and *SERPINA1* (encoding α1-antitrypsin which inhibits cleavage of the reactive center loop that releases cortisol from corticosteroid binding globulin). *SERPINA1* and *SERPINA*6 genes are both highly expressed in tissues associated with cortisol physiology, such as the liver and pancreas, supporting association between SNPs and plasma cortisol levels (10). These 3 partially correlated SNPs (linkage disequilibrium R^2^ ranged from 0.074 to 0.265 in European populations) were identified in a genome-wide association study (GWAS) meta-analysis for morning plasma cortisol levels in 12 597 participants of European ancestries and replicated in 2795 participants (11).

Summary-level GWAS data for the associations between the cortisol-associated SNPs and AF were obtained from a GWAS meta-analysis of 6 studies (The Nord-Trøndelag Health Study, deCODE, the Michigan Genomics Initiative, DiscovEHR, UK Biobank, and the AFGen consortium) with a total of 60 620 AF cases and 970 216 noncases of European ancestries (12) and the FinnGen consortium with 17 325 AF cases and 97 214 noncases of European ancestries (13). Summary-level GWAS data for systolic blood pressure and waist circumference were obtained respectively from the International Consortium of Blood Pressure (n = 757 601 participants) (14) and Genetic Investigation of ANthropometric Traits consortium (n = 232 101 participants) (15). Ethical approval and informed consent from participants had previously been obtained in individual studies included in the GWAS meta-analyses.

### Power Calculations

A power calculation was performed using the mRnD software (16). The significance level was set at .05 and the proportion of variation in cortisol explained by SNPs (R^2^) was 0.54% (11). Due to the lack of previous evidence explicitly assessing the association of hypercortisolemia with AF risk, we used the average estimates from 2 large-scale investigations into the effect of chronic psychological stress on AF, which resulted in an average odds ratio (OR) of 1.25 (8, 9). Under these assumptions, 99% power was estimated for the GWAS meta-analysis and 58% power for FinnGen alone (100% in the pooled sample).

### Statistical Analysis

The MendelianRandomization package for the R software was used for the statistical analyses (17). The fixed-effects inverse-variance weighted model with adjustment for the correlations among SNPs was used as statistical method. The correlation matrix was obtained in 367 643 unrelated participants of European ancestries in UK Biobank. Multivariable MR analysis was conducted to assess potential mediating effect of systolic blood pressure and waist circumference. The MR estimates derived from analysis of the 2 data sources were combined using fixed-effects meta-analysis.

## Results

**Figure 1.**
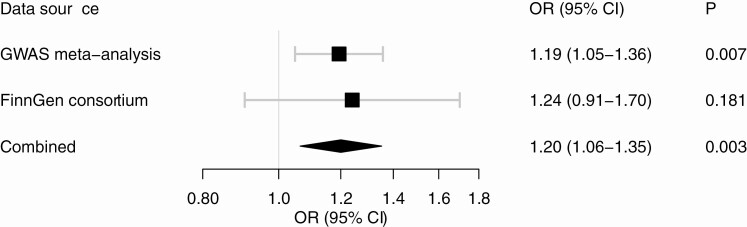
Association between genetically proxied plasma cortisol and risk of atrial fibrillation. The estimates are scaled per 1 SD increase in plasma cortisol and were derived from the fixed-effects inverse-variance weighted method with adjustment for the correlations between genetic variants.

**Figure 2.**
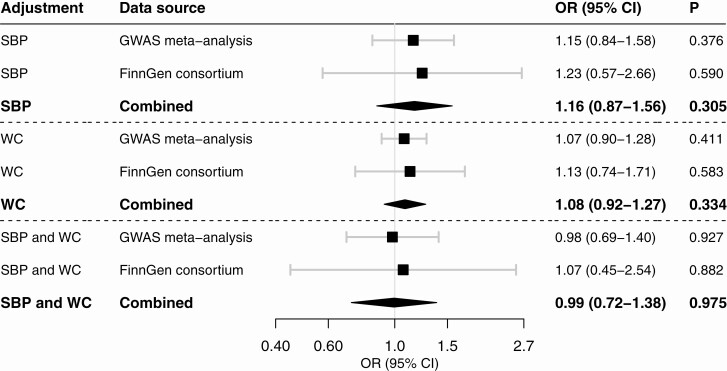
Association between genetically proxied plasma cortisol and risk of atrial fibrillation in multivariable Mendelian randomization analysis adjusted for genetically predicted systolic blood pressure (SBP), waist circumference (WC), or both. The estimates are scaled per 1 SD increase in plasma cortisol and were derived from the fixed-effects inverse-variance weighted method with adjustment for the correlations between genetic variants and for SBP, WC, or both.

The 3 SNPs used to proxy plasma cortisol and their associations with AF in the 2 data sources are shown in Table 1. Higher genetically proxied plasma cortisol levels were associated with a statistically significant increased risk of AF in the GWAS meta-analysis, and the MR estimate was of similar magnitude but nonsignificant in the FinnGen consortium (Fig. 1). In meta-analysis of results from the 2 data sources, the OR of AF per 1 SD increase of plasma cortisol was 1.20 (95% CI 1.06-1.35). The association between genetically proxied plasma cortisol and AF was attenuated in multivariable MR analysis with adjustment for genetically predicted systolic blood pressure or waist circumference, and did not persist after adjustment for both mediators (OR 0.99, 95% CI 0.72-1.38) (Fig. 2).

**Table 1.** Characteristics of the single nucleotide polymorphisms used to proxy plasma cortisol levels and their associations with AF

				Plasma cortisol		AF in GWAS meta-analysis^*a*^			AF in FinnGen		
SNP	Chr	Gene	EA	Beta (SE)^*b*^	*P*	Beta	SE	*P*	Beta	SE	*P*
rs12589136	14	*SERPINA6*	T	0.10 (0.015)	3.3 × 10^–12^	0.011	0.008	.175	0.016	0.020	.418
rs11621961	14	*SERPINA6*	T	–0.08 (0.013)	4.0 × 10^–8^	–0.017	0.007	.018	–0.019	0.017	.272
rs2749527	14	*SERPINA1*	T	–0.08 (0.013)	5.2 × 10^–11^	–0.021	0.007	.002	–0.024	0.016	.143

Abbreviations: AF, atrial fibrillation; Chr, chromosome; EA, effect allele; GWAS, genome-wide association study; SE, standard error; SNP, single nucleotide polymorphism.

^*a*^Includes data from 6 studies, including The Nord-Trøndelag Health Study, deCODE, the Michigan Genomics Initiative, DiscovEHR, UK Biobank, and the AFGen Consortium.

^***b***^The beta coefficients and corresponding standard errors represent the age- and sex-adjusted cortisol z-score change in morning plasma cortisol per additional effect allele in 12 597 participants of European ancestries.

## Discussion

This MR study is the first to utilize genetic variants associated with cortisol to examine causal association with AF. Results showed that genetic predisposition to higher plasma cortisol is associated with an increased risk of AF. Elevated cortisol levels in CS are associated with hypertension and central obesity (2-4), both of which result in left ventricular hypertrophy and structural changes (18, 19) that increase the risk for AF (20-23). In this study, the association between genetically predicted cortisol levels and AF appeared to be mediated by systolic blood pressure and waist circumference, as the association attenuated upon adjustment for these factors.

This study has several strengths. Compared with observational studies, MR is less prone to biases such as residual confounding and reverse causality. In an observational context, cortisol could increase AF susceptibility, but AF could also lead to increased internal stress that heightens circulating cortisol levels. This is unlikely when using genetic instruments, as these are fixed at conception. Secondly, it is worth noting that the availability of genetic variants in *SERPINA1* and *SERPINA6*, implicated in cortisol transport via corticosteroid binding globulin, enabled us to use highly specific MR instruments. Approximately 80% to 90% of circulating cortisol is bound to corticosteroid binding globulin, which plays a critical role in regulating plasma cortisol levels and modulating tissue availability of biologically active free cortisol (24, 25). Thirdly, the slowly progressive, nonspecific set of symptoms for CS that converge and coexist with common chronic diseases contributes to profound underreporting and underdiagnosis of this disease in the general population. Finally, because MR approximates long-term, cumulative cortisol exposure over a lifetime, the findings of this study can not only encourage AF monitoring in patients with CS, but also provide insight into potential risk of AF caused by sustained hypercortisolemia in general populations.

This investigation has however a number of limitations. Firstly, the SNPs selected for the present study only explain 0.54% variation in morning plasma cortisol. In theory, our results may be susceptible to the weak instrument bias, distorting estimates towards the null. However, our preference for SNPs located in a biologically relevant locus reduces this possibility. Secondly, results of the MR study could also be confounded if any our selected SNPs were in linkage disequilibrium with other correlated SNPs causally associated to AF via a noncortisol-mediated pathway (26, 27). Since linkage disequilibrium (LD) typically affects only nearby genetic variants, our analyses mitigate the risk of LD by modelling genetic effect on cortisol via multiple variants (27). Additionally, if multiple variants in LD are used as independent genetic exposures, genetic effects on cortisol will be fundamentally overestimated. Though there may be a small degree of LD between the 3 SNPs used in this study, they also show different associations with corticosteroid binding globulin biochemistry (11), suggesting that they exert independent effects on cortisol. Thirdly, the genetic effects of cortisol might be buffered by a physiological compensatory mechanism regardless of the underlying genotype, a phenomenon termed canalization that can lead to bias in the genetic associations with cortisol (28). Since cortisol is highly regulated, compensatory processes can turn on in response to perturbations of cortisol homeostasis, resulting in underestimated genetic effects. Finally, our datasets primarily include only European cohorts which limits applicability of study results to non-European populations.

Despite these limitations, our findings highlight the potential relevance of hypercortisolemia to AF and provide mechanistic insight into this association. Overall, these results are supportive of screening for AF in patients with CS, and suggest that the impact of prolonged hypercortisolemia on AF in general populations should be investigated more closely.

## Data Availability

All data used in this study are publicly available summary-level data, with the relevant studies cited. Data needed for the primary analyses are available in Table 1.

## Abbreviations

AF: atrial fibrillation
CS: Cushing’s syndrome
GWAS: genome-wide association study
LD: linkage disequilibrium
MR: Mendelian randomization
OR: odds ratio
SBP: systolic blood pressure
SNP: single nucleotide polymorphism
WC: waist circumference

## Additional Information

***Disclosures:*** The authors have no conflicts of interest to declare.
